# Content appraisal and age moderate the relationship between passive social media use and mental ill-being

**DOI:** 10.3389/fpsyg.2023.1181233

**Published:** 2023-07-14

**Authors:** Olivia Evans, Stephanie Hardacre, Mark Rubin, Max Tran

**Affiliations:** ^1^School of Medicine and Psychology, The Australian National University, Canberra, ACT, Australia; ^2^Department of Psychology, Durham University, Durham, United Kingdom; ^3^School of Psychology, The University of Newcastle, Callaghan, NSW, Australia

**Keywords:** Facebook use, passive social media use, mental ill-being, user age, subjective content appraisal, positive valence

## Abstract

An important distinction to make when assessing the impact of social media use on mental health is whether the use is passive (e.g., browsing) or active (e.g., posting). Recent research suggests that the connection between passive social media use and mental ill-being is inconsistent, with some research finding a significant negative association, while other research finds no such association. In the present research, we sought to investigate this relationship, as well as two potential moderators of this relationship: the subjective appraisal of social media content social media users consume (i.e., positively or negatively-appraised) and age of users. In a cross-sectional survey of Australian and United States Facebook users (*N* = 991), there was no direct relationship between passive use and mental ill-being, however user age and positive (but not negative) content appraisal were found to moderate the relationship between passive use and mental ill-being. Specifically, the relationship between passive use and mental ill-being became weaker as subjective positive appraisal increased, and it reversed to become negative at high levels of positive appraisal. Additionally, the positive relationship between passive use and mental ill-being became weaker as age of social media users increased, and the direction of this relationship became negative at the oldest ages of social media users. These results suggest that the relationship between social media use and mental ill-being is more nuanced than previous research suggests. In particular, higher amounts of passive Facebook use may have a less negative, or even a positive effect on social media users’ mental health when the content being (passively) consumed is positively appraised, or when users are older.

## Highlights

- Investigated content appraisal and user age as moderators of passive social media use and mental health.
- Exposure to positively appraised content buffered the negative impact of passive use on mental ill-being.
- Relationship between mental ill-being and passive use only had negative impact on younger participants.
- Passive use associated with better mental health for older participants.
- The impact of passive use is determined by who is undertaking it and how the content they are engaging with is appraised.

## Introduction

Facebook and other social networking sites (SNS) are intended to make us feel more connected to our social support systems, and to expand our social networks wider than ever before. However, the prevailing narrative about these sites is they are actually making us less connected to one another and are negatively impacting our mental health (Verduyn et al., 2021). Despite these largely negative accounts of SNS, research on the actual impact of social media is mixed. For example, some research suggests certain types of social media use are beneficial for well-being and social connectedness, while other research points to social media’s negative impacts, including rising rates of upward social comparisons and alienation (Steers, 2016; Frost and Rickwood, 2017; Verduyn et al., 2017).

Importantly, in recent decades there has been growing consensus that mental well-being and ill-being are not two sides of the same coin, but rather two distinct continua of mental health (Meier and Reinecke, 2021). For instance, while typical subjective well-being indicators include life satisfaction, happiness and positive affect, ill-being indicators include depressive symptoms and negative affect (i.e., anxiety and stress). While both well-and ill-being are associated with social media use, in the present paper we focus solely on ill-being because well-being (as well as the depression aspect of ill-being) has received ample attention, while ill-being indicators such as anxiety and stress remain underexplored (Verduyn et al., 2017; Keles et al., 2020). Several recent reviews have concluded the association between Facebook use and both mental well-and ill-being is relatively complex and nuanced (Steers, 2016; Frost and Rickwood, 2017; Verduyn et al., 2017; Yin et al., 2019). These reviews suggest the *way* in which people are using Facebook is one of the key factors in determining its impact on mental health. Specifically, they distinguish between passive social media use (PSMU), whereby users simply observe others’ posts, and active social media use, whereby users generate their own material and updates, and share and comment on others’ posts (Steers, 2016; Verduyn et al., 2017).

In terms of negative consequences, some research has found PSMU has a distinct negative effect on mental well-and ill-being, especially when compared to active use (Tandoc et al., 2015; Frison and Eggermont, 2016; Verduyn et al., 2017). For example, Frison and Eggermont (2016) discovered that passive Facebook use positively predicted adolescents’ depressed mood. The dominant theory behind this finding is that PSMU leads to upward social comparisons that negatively impact on well-being due to reducing users’ positive feelings and self-esteem, increasing feelings of envy, and making users feel worse about themselves (Verduyn et al., 2017). Alternatively, active social media use is thought to increase well-being by prompting positive feedback and support from others. However, a scoping review conducted by Valkenburg et al. (2021) revealed inconclusive evidence regarding the adverse effects of PSMU on mental well-and ill-being. While the effects of PSMU received stronger support for ill-being compared to well-being outcomes, this was only supported by a minority of studies (i.e., 15 out of 34) and thus the evidence remains limited. These findings further strengthen our decision to concentrate on and explore ill-being outcomes in the current paper. Specifically, based on previous work (e.g., Frison and Eggermont, 2016) we predict that PSMU will be negatively associated with mental ill-being.

Together with Valkenburg et al.’s review, emerging research on social media use suggests a more nuanced approach is needed when investigating PSMU and its bearings on mental well-and ill-being. In particular, researchers have suggested the objective *content* (e.g., positive vs. negative) users consume on social media may be more important than whether their social media use is passive or active (Valkenburg et al., 2021). Additionally, more needs to be understood about why the findings regarding the impact of PSMU on mental ill-being are inconsistent, which may be due to as yet unexplored moderators of this relationship. Specifically, Valkenburg et al. (2021) suggest that future research “should take characteristics of the … receivers (e.g., differential susceptibility) into account” (p. 1). As such, in the present paper we consider two potential moderators of the relationship between PSMU and mental ill-being: subjective appraisal (or perceived valence) of the content users consume and user age.^1^ Note that the hypotheses reported hereafter were generated after collecting the data and discovering that PSMU was not significantly related to mental health as originally hypothesized. Below, we outline and justify these exploratory hypotheses based on existing research and theory.

### Content appraisal

As suggested by Valkenburg et al. (2021) the content of social media is likely a more important deciding factor in the relationship between social media use and mental health, than the type of use in itself (i.e., active vs. passive). Facebook, and social media more broadly, covers a wide span of user-generated content that can vary from being positive, negative or decidedly neutral in substance. The appraisal of content may then be an important consideration in the impact that social media has on mental health and well-being, as research has demonstrated viewing positive images and affirming messages boosts well-being and improves mental health via selectively activating those brain structures associated with pleasure and reward processing; whereas exposure to negative content such as cynical or confrontational messages has a deleterious effect on mood and well-being (Holmes and Mathews, 2010; Palacios, 2018). In terms of social media in particular, consumption of positive content (e.g., pictures of puppies) can also provide greater potential for increased social support and social connectedness for users, both of which contribute to mental health (Verduyn et al., 2017).

Interestingly Meier et al. (2020) experimentally found exposure to “inspiring” Instagram travel posts resulted in increases in users’ positive affect. In contrast, Krasnova et al. (2015) discovered travel and leisure posts—such as those relating to vacations, concerts, and free time—elicit more negative reactions and envious feelings compared to other content, such as that pertaining to relationships, success, and appearance. Moreover, these feelings of envy arising from consumption of fellow Facebook friends’ social information were associated with decreased affective well-being among Krasnova et al.’s sample of college-age Facebook users. Importantly, they did not differentiate between individual users’ *interpretations* (i.e., subjective appraisal) of the travel and leisure content—for example whether the participants found these posts to be inspiring, pretentious, or otherwise. These contrasting findings speak to the subjective appraisal *induced by* the content, rather than the objective content *itself* (i.e., content valence), playing a moderating role in the relationship between PSMU and users’ well-being.

Hence, one reason for the mixed evidence regarding the impact of PSMU on mental health could be that researchers have not accounted for the *appraisal* of the content people are viewing. Consequently, in the present research we take current investigations of social media content a step further by examining the impact the *subjective (or perceived) appraisal* of the content users consume has on their mental ill-being, rather than examining the objective content itself. In this sense, we focus on the emotions and feelings induced by the consumed content, which can differ significantly between individuals based on their interpretation (i.e., appraisal) of said content. For example, social media users experiencing infertility may perceive their Facebook friend’s pregnancy announcement as being negatively valenced given the feelings of envy, bitterness and sadness such an emotional trigger might induce (Collins, 2018). Meanwhile, users prone to indulging in schadenfreude might appraise their academic rival’s latest article rejection on Twitter as inherently positively valenced due to the (albeit malicious) joy and pleasure they feel seeing their rival’s visible misfortune, leading to an increase in the user’s self-worth and well-being (Cecconi et al., 2020). In this sense, it could be expected the passive consumption of seemingly “objectively” positive and/or negative social media content could have vastly different impacts on users’ mental well-and ill-being as a function of their perceived appraisal (i.e., subjective “valence”) of that content.

Based on the above, we argue that there will be a negative association between passively consumed content that is appraised positively and mental ill-being scores, such that participants who passively consume positive content will report lower mental ill-being. We additionally argue that the relationship between PSMU (in particular, passive Facebook use) and mental ill-being could be moderated by subjective content appraisal, such that the negative relationship between PSMU and ill-being becomes weaker when the content being consumed by the user is appraised by them as being positive. In contrast, the opposite effect would occur for content appraised as negative. Specifically, when the content being consumed by the user is appraised as being negative, the negative relationship between PSMU and mental ill-being should be stronger.

### Age

Another potential moderator of the relationship between PSMU and mental health is user age. Social ties and interactions differ across different age groups and generations (Akiyama et al., 2003; Birditt et al., 2009), and researchers have begun to investigate the translation of these age differences into online interactions (Hayes et al., 2015; Francis, 2018; Sharifian and Zahodne, 2020). However, most work to date has focused on adolescents, young adults, or had a relatively young sample overall (Twenge et al., 2018; Coyne et al., 2020). This is problematic in light of recent research, which has found the relationship between general social media use and well-being is moderated by age (Sharifian et al., 2021), such that the positive relationship between social media use and depressive symptoms is present for younger but not older participants. However, this research did not differentiate between different kinds of social media use (e.g., active vs. passive) or delve further into this moderation relationship.

This is an important gap to address, given the ways people engage with social media, and the impact its use has on their well-being, is likely to change with age. For example, as we progress through adolescence into adulthood we develop more proactive and enriching socialization skills and behaviors (Samter, 2003). In general, younger people are more sensitive to antisocial or mood-affecting social behaviors such as social comparison than are older adults (Altikulaç et al., 2019). In their 2015 study of 18–24 year old Facebook users, Krasnova et al. used social comparison theory as a basis to investigate the role of envy as a potential moderator of the negative impacts of social media on mental health. They concluded young adults may be especially vulnerable to social media-induced envy (and associated reductions in affective well-being) given their “developing identities, the importance they attach to social relationships, [and] uncertainties they face about the future” (Altikulaç et al., 2019, p.587) compared to older adults.

There are also generational differences in people’s exposure to social media, with younger people viewing and using social media as an extension of their everyday lives, whereas older generations view it as a distinct social tool separate to their offline selves (Spies Shapiro and Margolin, 2014; Chang et al., 2015; Wood et al., 2016). Older generations thus tend to be more intentional and insightful in the way they engage with social media (Reed et al., 2014; Chang et al., 2015; Hayes et al., 2015), whereas Krasnova et al. (2015) cite the “always online” (p. 1,019) habit among young adults as potentially worsening negative mental health impacts social media use can have. Consequently, we expect there are age differences in the way PSMU impacts users’ mental health and well-being, such that older users will be less negatively impacted by PSMU than younger users. This is because even when older people engage in inherently *passive* use, it is likely to be in a more mindful, positive, and beneficial way compared to younger people. Indeed, negative mental health impacts of PSMU may be pronounced for younger people given their vulnerability to social comparison, feelings of envy, and ubiquitous social media use.

Based on this, we predict that the relationship between PSMU and mental ill-being should be moderated by social media user age, whereby the negative relationship between PSMU and ill-being becomes weaker when the age of the user is older. We expect the opposite effect to occur for younger social media users, whereby when the user is younger, the negative relationship between PSMU and ill-being should be stronger.

In the present research we conducted a survey of users’ social media use and mental health to investigate the relationship between PSMU and mental ill-being. We expected that PSMU would be positively associated with mental ill-being, such that as passive use increased so too did mental ill-being (i.e., poorer mental health). We also expected that the subjective appraisal of passively consumed content would impact mental ill-being. Specifically, we predicted that passively consumed content that was appraised positively would be negatively associated with mental-illbeing (i.e., lower mental ill-being scores). We additionally explored moderators of users’ PSMU, and the impact these moderators have on their mental ill-being. Specifically, we examined the potential for subjective content appraisal and user age to moderate the relationship between users’ PSMU and ill-being. We expected this relationship to become weaker as positive subjective appraisal and age increased, and stronger as negative subjective appraisal increased and age decreased.

## Materials and methods

### Participants and procedure

Participants were 962 general population Facebook users, recruited through university participant pools and MTurk. We focus solely on Facebook due to the existing research concentrating on its usage, allowing us to extend and build upon these findings. Facebook is also one of the most widely used social networking sites, with around half of the current online population having an account, and an estimated 90% of higher education students being users (Dahlstrom et al., 2011). The student participants were from a large regional Australian university (*n* = 460), and the MTurk workers were from the USA (*n* = 502). Across the samples there were 482 males and 478 females, with ages ranging from 17 to 97 years (*M* = 31.10, *SD* = 13.15). A breakdown of the demographics for each of these samples can be found in Supplementary material. To account for differences between the two convenience sampling pools, source of participants was included as a covariate in all reported analyses, with the pattern of results remaining unchanged.

### Measures

Participants took part in a 10–15 min online survey, where they completed measures relating to their social media use and mental health, before completing an imagination task focused on gender. Since the measures reported in this paper were assessed prior to the completion of this task, it is not pertinent to the results presented here. Full details about the survey can be found in the preregistration for this project at this link. For brevity, we only discuss the specific survey items relevant to the present research.

Mental ill-being was measured using Lovibond and Lovibond’s (1996) 21-item Depression Anxiety and Stress Scale (DASS-21), which is designed to measure frequency and severity of depression, anxiety and stress symptoms. Participants responded to each item on a 4-point Likert scale, ranging from 0 (*never*) to 3 (*almost always*). All items were summed within their subscales (7 items each) to create separate measures of depression, anxiety and stress, as well as summed together to create composite measure of general mental ill-being.

Based on previous research (Wenninger et al., 2014; Verduyn et al., 2015), we developed our own scale to measure participants’ frequency of passive Facebook use. The initial scale contained 18 items regarding how frequently participants engaged in different Facebook behaviors (e.g., reading, liking posts, etc.). Participants responded to each item using a 7-point Likert scale, with response options ranging from 1 (*almost always*) to 7 (*very rarely*).^2^ Based on exploratory factor analysis results, nine types of passive use behaviors were included in the final scale, including reading posts on the newsfeed, pages and in groups; reading comments on posts; watching videos; viewing pictures; looking at profiles; and reacting to/liking posts and comments.^3^ Scores from the passive use questions were averaged to form an aggregate score of passive use frequency.

Subjective content appraisal was measured using two scales that assessed *subjective valence* of the content participants consumed on Facebook. For each scale, we developed 13 items reflecting common types of positive and negative valenced content people might view on Facebook. Participants were asked “Please indicate the extent to which you agree or disagree that each of the words below describes the material that you *view* on Facebook….” The positive items included “encouraging,” “friendly,” “inspirational,” “informative,” “light hearted,” and “supportive”; while the negative items included “cynical,” “hostile,” “confrontational,” “provocative,” “controversial,” “argumentative,” and “negative.” Each scale was presented twice, with reference to whether the content consumed and appraised by participants was interacted with passively (i.e., viewed) or actively (i.e., posted, commented on, reacted to). Scores from the positive and negative subscales were averaged to form separate scores of positive and negative valence (based on participants’ own subjective appraisal). In the present study only items relating to passive (and not active) use are discussed given our focus on the impact of PSMU.

## Results

### Preliminary analyses

Skewed variables were identified as those exceeding +/− 2.00, as specified in the preregistered document. Age was highly skewed and thus was log10 transformed for all analyses. The means, standard deviations, Cronbach’s alpha values and correlations of each of the variables are in Table 1. On average, participants visited Facebook 7.21 times each day (*SD* = 5.73) and had 254 Facebook friends (*SD* = 136.00). Participants also reported Facebook was open on their device for an average of 4.04 h per day (*SD* = 5.21).

**Table 1:** Descriptive statistics.

Measure	*M*	*SD*	Min	Max	α	1	2	3	4	5	6	7
1. Mental ill-being	14.82	13.12	0.00	63.00	0.96	-						
2. Depression	4.86	5.11	0.00	21.00	0.96	0.92^**^	-					
3. Anxiety	3.97	4.45	0.00	21.00	0.89	0.92^**^	0.76^**^	-				
4. Stress	5.99	4.69	0.00	21.00	0.89	0.92^**^	0.75^**^	0.80^**^	-			
5. Passive use	4.8	1.17	1.00	7.00	0.93	−0.01	−0.04	−0.01	0.04	-		
6. Positive appraisal	5.08	1.08	1.00	7.00	0.85	−0.08^*^	−0.11	−0.03	−0.06	0.29^**^	-	
7. Negative appraisal	4	1.42	1.00	7.00	0.92	0.34^**^	0.29^**^	0.30^**^	0.34^**^	0.07^*^	−0.21^**^	-
8. Age^t^	1.46	0.16	1.23	1.99	-	−0.08^*^	−0.04	−0.07^*^	−0.11^**^	−0.06	0.15^**^	−0.11^**^
9. Age	31.1	13.15	17.00	97.00	-	−0.05	−0.02	−0.04	−0.07^*^	−0.06	0.15^**^	−0.09^**^

*N* = 962 for all variables reported above. t indicates variables that have been log10 transformed. ^*^p < 0.05, ^**^p < 0.001.

Pearson’s correlations showed that inconsistent with previous research and with our prediction, passive use was not significantly negatively associated with mental ill-being or the separate subscales of depression, anxiety and stress. Consistent with our prediction, positive appraisal of passively viewed content was significantly negatively correlated with ill-being, such that participants who evaluated passively viewed content more positively had lower scores of mental ill-being and depression but not anxiety and stress. In contrast, negative appraisal of passively viewed content was significantly positively correlated with ill-being as well as depression, anxiety and stress. Age was weakly positively associated with ill-being, anxiety and stress but not depression, while age was not significantly correlated with passive use.

### Positive and negative subjective content appraisal as moderators

**Figure 1:**
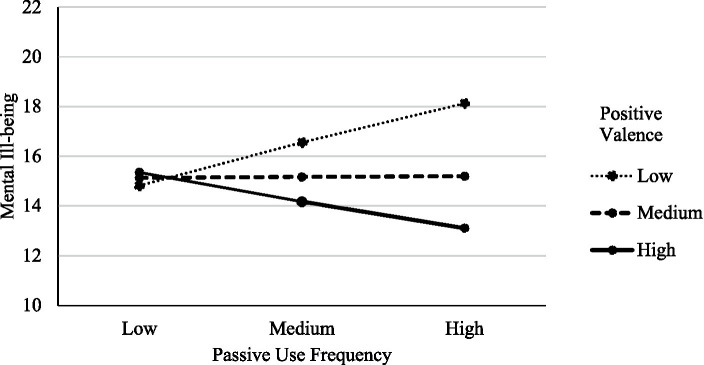
The relationship between PSMU frequency and mental ill-being as a function of level of positively valanced content.

We tested the hypothesis that type of subjective content appraisal would moderate the relationship between passive Facebook use and ill-being using Hayes (2017) PROCESS model 1. All variables were centered prior to analysis. The results did not depict negative appraisal as a significant moderating factor in the relationship between passive Facebook use and mental ill-being, *b* = −0.18, *SE* = 0.81, *t*(957) = −0.82, *p* = 0.859. However, as expected, the relationship between passive Facebook use and ill-being was significantly moderated by positive appraisal, *b* = −1.19, *SE* = 0.28, *t*(957) = −4.31, *p* < 0.001. The relationship between passive use and ill-being was positive and significant at lower levels of positive appraisal *b* = 1.08, *SE* = 0.47, *t*(957) = 3.02, *p* = 0.003, 95% CI (0.50, 2.33). This relationship was not significant at the mean levels of positively appraised content, *b* = 0.01, *SE* = 0.38, *t*(957) = 0.38, *p* = 0.940, 95% CI (−0.71, 0.77); however, the relationship was negative and significant at higher levels of positively appraised content, *b* = −0.92, *SE* = 0.46, *t*(957) = −2.09, *p* = 0.037, 95% CI (−1.87, −0.06). Hence, the relationship between passive use and ill-being became weaker as positive subjective appraisal of content increased, and it reversed to become negative at high levels of positive appraisal. Figure 1 provides a visual representation of this moderation effect. Table 2 includes the results from this same moderation model with the separate subscales of depression, anxiety and stress as outcomes. The direction and significance of this moderation model remained the same as the aggregate measure for all the subscales of the DASS.

**Table 2:** Moderation results with separate depression, anxiety and stress subscales.

**X = passive use M = negative appraisal**	**b**	**SE**	**df**	**t**	**p**	**99% CI**	
Depression (X × M)	0.00	0.89	4, 957	−0.05	0.961	−0.18	0.17
Anxiety (X × M)	−0.12	0.08	4, 957	−0.16	0.11	−0.27	0.03
Stress (X × M)	−0.06	0.08	4, 957	−0.72	0.47	−0.22	0.10
**X = passive use M = positive appraisal**	**b**	**SE**	**df**	**t**	**p**	**99% CI**	
Depression (X × M)	−0.37	0.11	4, 957	−3.5	0.001	−0.58	−0.16
Anxiety (X × M)	−0.43	0.09	4, 957	−4.58	<0.001	−0.61	−0.25
Stress (X × M)	−0.34	0.1	4, 957	−3.92	<0.001	−0.58	−0.19
**X = passive use M = Age^t^**	**b**	**SE**	**df**	**t**	**p**	**99% CI**	
Depression (X × M)	−2.56	0.88	4, 948	−2.91	0.004	−4.29	−0.83
Anxiety (X × M)	−2.64	0.77	4, 948	−3.43	0.001	−4.15	−1.13
Stress (X × M)	−3.46	0.39	4, 948	−4.3	<0.001	−5.05	−1.88

t indicates variables that have been log10 transformed.

### Age as a moderator

**Figure 2:**
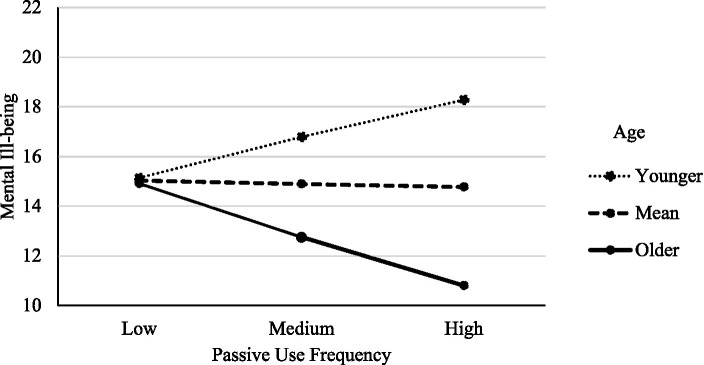
The relationship between PSMU frequency and mental ill-being as a function of level of user age.

We then tested the hypothesis that age would moderate the relationship between passive Facebook use and mental ill-being. As expected, the relationship between passive Facebook use and ill-being was significantly moderated by age, *b* = −8.67, *SE* = 2.26, *t*(948) = −3.83, *p* < 0.001. The relationship was positive and significant at younger ages *b* = 1.34, *SE* = 0.53, *t*(948) = 2.52, *p* = 0.012, 95% CI (0.30, 2.39); nonsignificant at the mean age, *b* = −0.01, *SE* = 0.36, *t*(948) = −0.32, *p* = 0.749, 95% CI (−0.82, 0.59); and negative and significant at older ages, *b* = −1.76, *SE* = 0.55, *t*(948) = −3.19, *p* = 0.002, 95% CI (−2.84, −0.68). Hence, consistent with hypotheses, the positive relationship between passive use and ill-being became weaker as age increased, and the direction of this relationship became negative at the oldest ages. Figure 2 provides a visual representation of this moderation effect. Table 2 includes the results from this same moderation model with the separate subscales of depression, anxiety and stress as outcomes. The direction and significance of this moderation model remained the same as the aggregate measure for all the subscales of the DASS.

### Outlier exclusion

We reconducted all analyses excluding univariate outliers and including control variables (source of participants and gender). This did not alter the pattern of results. Given the mean age was significantly higher in the MTurk sample (*M* = 38.30, *SD* = 13.43) than the university sample (*M* = 23.55, *SD* = 7.81; *t*(980) = −20.89, *p* < 0.001) we also tested source of participants as a moderator in place of age (e.g., predictor = passive use, moderator/mediator = source of participants, outcome = mental ill-being). Importantly, this result did not produce a significant moderation effect, suggesting it is not differences in the sampled participant pools driving the observed moderating effect of age.

## Discussion

As predicted, participants who appraised passively consumed social media content more positively had lower scores of mental ill-being. Additionally, as expected, the relationship between passive Facebook use and mental ill-being was significantly moderated by level of positively appraised content and user age. However, contrary to predictions, negatively appraised content was not found to be a significant moderator of the relationship between passive Facebook use and mental health. Overall, our results support the idea that the relationship between PSMU and mental well-being is more nuanced than has thus far been proposed in the literature.

Notably, contrary to our predictions, there was no direct relationship between PSMU and mental ill-being. While inconsistent with findings of some other studies (Tandoc et al., 2015; Frison and Eggermont, 2016; Verduyn et al., 2017), this is consistent with more recent findings that have found ambiguous associations between PSMU and mental well-and ill-being (Valkenburg et al., 2021). A key reason for this lack of a direct relationship in the current study could be because our sample was older than those used in previous research. The moderation effect for age was such that the relationship was significant and positive for younger participants and significant but negative for older participants. Thus, the direct relationship between PSMU and mental health is canceled out by this interaction which had an amplification rather than buffering effect. Another explanation is we only included a mental ill-being measure (i.e., depression, anxiety, and stress), whereas some research has additionally focused on positive mental well-being (e.g., satisfaction with life, personal growth, etc.). Thus, it is possible PSMU has a direct relationship with mental *well*-being, and not *ill*-being. As such, future research could assess both dimensions of mental health.

### Content appraisal

The present study is also the first to consider the impact of the *subjective appraisal* of social media content consumed—that is, from the users’ own perspective—rather than merely considering the objective content itself (e.g., pictures of puppies). This focus on appraisal as opposed to content offers a novel perspective to the social media literature. Recall our previous example: a pregnancy announcement (i.e., objective content) will likely have a different subjective appraisal and subsequent impact on well-being for the passive social media user experiencing infertility (i.e., negative appraisal) vs. the user who is not (i.e., positive appraisal). As predicted, passively consumed content that was positively appraised was associated with lower mental ill-being scores (i.e., better mental health). Moreover, as expected, the relationship between mental ill-being and PSMU was weaker when appraisal was more positive, suggesting PSMU has less of an impact on mental ill-being when content is perceived as positive (e.g., encouraging, inspirational) rather than negative (e.g., hostile, cynical). Unexpectedly however, the reverse result was not found for negatively appraised content. Consequently, our findings provide support for seeking out subjectively positive content on social media to protect one’s mental health, but does not demonstrate similar benefits for avoiding subjectively negative content.

Though unexpected, this finding is in fact reassuring given how difficult it can prove for users to *avoid* subjectively negatively valenced content on social media, based on processes such as algorithmic content selection, algorithmic filtering, and user-generated social curation (Merten, 2021). In contrast, it is easier for users to intentionally *seek out* subjectively positively valenced content by curating their social media feed, providing themselves “a certain kind of leverage in determining its composition” (Merten, 2021, p. 1,019). The “personal curation practices” of social media users have garnered increased attention and traction more recently. Personal curation acts include following preferred media outlets and influencers, blocking or “hiding” content from certain Facebook friends, and choosing to include or exclude specific topics or opinions, all in a bid to either boost or limit exposure to particular content (Thorson and Wells, 2016; Merten, 2021). Relatedly, in the context of Covid-related social media news content, Buchanan et al. (2021) discovered exposure to just 2–4 min of doom-scrolling led to immediate reductions in positive affect, whereas “kindness-scrolling” (p. 1) did not. Based on our finding that positively appraised content weakened the relationship between passive Facebook use and ill-being, such curation practices may herald an effective way to proactively improve one’s well-being.

### Age

Finally, our findings provided support for the hypothesis that older age is a protective factor for the association between PSMU and mental ill-being. These findings support previous research which has found age group differences in the impact of social media use on socioemotional health may be driven by both generational effects and aging effects relating to socioemotional development (Sharifian et al., 2021). Our findings also provide evidence that the way people engage with social media and the effect its use has on mental health changes with age. Namely, in line with Krasnova et al. (2015), our results indicated younger users appear particularly vulnerable to negative mental health impacts associated with passive Facebook use. Indeed, Krasnova and colleagues theorized that “envy processes and the associated undesirable outcomes [of social media use] may be especially pronounced for younger users because they have limited coping experience” (p. 600). This tracks with differences in generational effects and socioemotional development between younger and older users. Our findings regarding the mental health impacts of social media use are particularly pertinent because young adult users are a distinct population group given their near ubiquitous adoption of social media (Krasnova et al., 2015).

Further research is needed to determine the reason for these age effects. As touched on, these differences might be due to generational and/or age differences in socialization skills, exposure to social media, and/or different intentions or reasons for using social media. Establishing which of these aspects offers the best explanation of these age differences will be instrumental in determining how these findings can be applied to reduce the harmful mental health impacts of social media use.

### Limitations and future directions

The present study offers several strengths compared to previous research in the area. In particular, the samples obtained are from two countries (Australia and the United States), and cover a broader age range than prior research on PSMU and well-being. Thus, as well as being the first to investigate potential moderators of PSMU and mental health, this study is the first to investigate these effects across a wider range of individuals. However, it should be noted that despite the increase in sample size and demographics, the present study used two different convenience samples (i.e., students and online crowdsourcing), which is not ideal in terms of confidence in the generalizability of our results. The sensitivity analyses including participant source make us confident the findings are not attributable to differences in the kinds of participants sampled. Nonetheless, future research should endeavor to obtain a more naturalistic sample.

This research also focused exclusively on Facebook use. Although Facebook is among the most popular social networking sites, various sites are associated with diverse histories and differing patterns of use and user characteristics (Wilson et al., 2012). Thus, future research should seek to gain a broader picture of social media use to ensure these findings are not limited to one platform and have longevity over time as technology develops (e.g., social media algorithms). Moreover, the present research relied on self-reported social media use. Users, particularly young adults, often underestimate the time spent engaging with social media (Ernala et al., 2020) which may impact accuracy of the present findings. This is especially relevant to the current study given the already huge amount of time our sample reported spending on social media (i.e., upwards of 4 h per day), indicating they are “chronically online.” Despite self-report being necessary to obtain participants’ subjective appraisals of the valence of content, future research should attempt to address this issue by additionally using observational or objective social media use measures. For example, iPhone screen tracking features can reliably collect objective screen time data (Hodes and Thomas, 2021). Longitudinal designs would also allow better tracking of users’ changes in PSMU over time and associated impacts on their mental health, for example by asking participants to recall the subjective appraised valence of the content they consumed and how they felt directly after consuming it.

Finally, while valid, the way our self-constructed subjective appraisal instrument was operationalized means it likely captures only a small fraction of all content available on social media. This research was only an initial conceptualization and test of our content appraisal theory, thus further work is needed to develop a stronger measure. For example, in a content analysis of user-generated descriptions of emotions arising from viewing friend’s social information on social media, Krasnova et al. (2015) coded two categories of positive (e.g., pleasure, admiration) and negative (e.g., envy, contempt) emotional states. These state descriptors arguably better capture subjective *emotional* appraisal that certain content on social media may induce compared to the more general adjectives we generated for our appraisal instrument that did not directly tap emotional states (e.g., encouraging, friendly, supportive vs. cynical, provocative, controversial). Moreover, there exists crossover with certain items on our positive and negative subscales. For example, a factor analysis suggested “informative” loaded onto the “positive” subscale, however this particular kind of appraisal could easily be considered negative tpp (e.g., in the context of war, sexual assault, etc.), and vice versa for our “provocative” item. Thus, while the present research demonstrates promise for the concept of subjective appraisal, future research would benefit from developing more specific emotional state descriptors in their operationalization of this concept.

### Conclusion

PSMU is a necessity when engaging in social media use, and thus determining the amplifiers and buffers of its effects on mental well-and ill-being are important in fostering more beneficial and enriching outcomes from social media. The present research suggests PSMU does not necessarily have to be a negative experience, but rather the impact of PSMU is determined by *who* is undertaking it and *how*. Specifically, our findings indicate consuming content appraised by the user as positive can improve mental well-being, and higher PSMU is more beneficial for older rather than younger individuals.

Our findings have implications for social network site providers and users alike. Firstly, providers may benefit from attracting more users by tweaking their algorithmic content selection and filtering processes to increase users’ exposure to content that is more personalized and more likely to be appraised positively by them. Secondly, our results call for increased caution regarding how younger users engage with Facebook and social media platforms more broadly. They specifically speak to the importance of users taking a more proactive approach to their PSMU. Namely, users would do well to harness the power of personal curation practices as a way to increase their exposure to social media content they perceive as positively valenced, and hopefully reap the mental health benefits of doing so. In this sense, kindness-scrolling could very well become the new doom-scrolling.

## Data availability statement

The raw data supporting the conclusions of this article will be made available by the authors, without undue reservation.

## Ethics statement

The studies involving human participants were reviewed and approved by University of Newcastle Human Research Ethics Committee. The patients/participants provided their written informed consent to participate in this study.

## Author contributions

OE: literature search, study design, recruitment, analysis and interpretation of the results, writing and revising drafts, and supplemental appendices. SH: literature search, interpretation of the results, and writing and revising drafts (including theoretical and practical feedback). MR: study design, recruitment, analysis and interpretation of the results, and revising drafts. MT: recruitment, analysis and interpretation of the results, and revising and formatting drafts. All authors contributed to the article and approved the submitted version.

## Funding

The work was supported by funding from the Australian Research Council (IN200100047). This research was completed as part of the MT’s honors thesis.

## Conflict of interest

The authors declare that the research was conducted in the absence of any commercial or financial relationships that could be construed as a potential conflict of interest.

## Publisher’s note

All claims expressed in this article are solely those of the authors and do not necessarily represent those of their affiliated organizations, or those of the publisher, the editors and the reviewers. Any product that may be evaluated in this article, or claim that may be made by its manufacturer, is not guaranteed or endorsed by the publisher.

## Footnotes

^1^The preregistration for the present project can be found on the Open Science Framework at https://osf.io/t62vp/?view_only=f6256d5cacbc469bb9685a480b8d3a2d. The preregistration details a number of planned hypotheses and analyses that are not reported in this article. The analyses reported in this article are based on hypotheses that were generated after collecting the data and discovering that PSMU was not significantly related to mental health, which is a premise that most of the preregistered hypotheses were based on. Thus, we generated a series of new hypotheses relating to moderators of this relationship based on existing research and theory, to shed light on these moderators and their associations with PSMU. Doing so contributes to the existing literature and provides a foundation for further investigation into this field that may inform the development of social media interventions and strategies to enhance mental health. For further details about this approach, please see Rubin (2020) and Rubin and Donkin (2022).

^2^This is the only scale in the survey that had reversed scale endpoints (i.e., with 1 *= almost always* and 7 *= very rarely*) with all other scales conforming to a format where 1 was the lowest point of the scale and 7 was the highest. This was unintentional and occurred due to a coding error during survey development process. Participants were only shown the text of the scale options and not the related code, however we acknowledge that the order of these responses may have made this scale less intuitive for the participants to answer.

^3^Based on the results of the exploratory factor analyses outlined in the pre-registration, liking and reacting to posts were included in the passive use scale.
