# Supplementary material for: Content appraisal and age moderate the relationship between passive social media use and mental ill-being
Source: Front Psychol. 2023 Jul 14;14:1181233. doi: 10.3389/fpsyg.2023.1181233 (PMC10388548; doi:10.3389/fpsyg.2023.1181233)
Supplement: Supplementary file 1 [file Table_1.DOCX]

**Supplementary Table 1**
*Demographics of each sampled participant pool*

| Variable | | Sona (N = 460) | | | MTurk (N = 502) | |
| --- | --- | --- | --- | --- | --- | --- |
|  | *N* | | Percentage | *N* | | Percentage |
| Gender |  | |  |  | |  |
| Women | 233 | | 50.7% | 245 | | 48.8% |
| Men | 227 | | 49.3% | 255 | | 50.8% |
| Self-described | 0 | | 0% | 2 | | 0.4% |
| Member of an ethnic minority |  | |  |  | |  |
| Yes | 62 | | 13.5% | Not available | |  |
| No | 398 | | 86.5% | Not available | |  |
| Ethnicity |  | |  |  | |  |
| White/Caucasian | Not available | |  | 351 | | 69.9% |
| Black/African American | Not available | |  | 74 | | 14.7$ |
| Hispanic or Latinx | Not available | |  | 36 | | 7.2% |
| Asian American | Not available | |  | 28 | | 5.6% |
| Native American | Not available | |  | 4 | | 0.8% |
| Self-described | Not available | |  | 9 | | 1.8% |
|  |  | |  |  | |  |

**Supplementary Table 3**
*Descriptive statistics for each sampled participant pool*

|  | Sona (N = 460) | | | |  | MTurk (N = 502) | | | |
| --- | --- | --- | --- | --- | --- | --- | --- | --- | --- |
| Measure | *M* | *SD* | Min | Max |  | *M* | *SD* | Min | Max |
| 1. Mental ill-being | 15.09 | 10.83 | 0.00 | 52.00 |  | 14.57 | 14.92 | 0.00 | 63.00 |
| 2. Passive use | 4.77 | 1.19 | 1.00 | 7.00 |  | 4.84 | 1.15 | 1.00 | 7.00 |
| 3. Positive valence | 4.94 | 1.01 | 1.00 | 7.00 |  | 5.21 | 1.13 | 1.00 | 7.00 |
| 4. Negative valence | 4.21 | 1.28 | 1.00 | 7.00 |  | 3.81 | 1.52 | 1.00 | 7.00 |
| 5. Age^t^ | 1.35 | 0.11 | 1.23 | 1.89 |  | 1.56 | 0.14 | 1.23 | 1.99 |
| 6. Facebook visits per day | 7.49 | 5.73 | 0.00 | 20.00 |  | 6.95 | 5.72 | 0.00 | 20.00 |
| 7. Hours Facebook open on device per day | 2.97 | 3.71 | 0.00 | 24.00 |  | 5.01 | 6.13 | 0.00 | 24.00 |
| 8. Facebook friends | 277.19 | 132.68 | 0.00 | 400.00 |  | 232.33 | 135.60 | 0.00 | 400.00 |

*Note.* t indicates variables that have been log10 transformed.
